# Cognitive behavioral therapy for insomnia among heavy-drinking veterans: a randomized pilot trial

**DOI:** 10.1093/sleepadvances/zpaf037

**Published:** 2025-06-10

**Authors:** Mary Beth Miller, Ryan W Carpenter, Sydney D Shoemaker, Katie R Moskal, Brian Borsari, Eric R Pedersen, Bruce D Bartholow, Douglas Steinley, Christina S McCrae

**Affiliations:** Department of Psychiatry, University of Missouri, Columbia, MO 65212, USA; Department of Psychological Sciences, University of Missouri, , Columbia, MO 65202, USA; University of Notre Dame, Notre Dame, IN 46556, USA; Department of Psychological Sciences, University of Missouri, , Columbia, MO 65202, USA; Department of Psychological Sciences, University of Missouri, , Columbia, MO 65202, USA; Mental Health Service (116B), San Francisco VAHCS, San Francisco, CA 94121, USA; University of California San Francisco, San Francisco, CA 94143, USA; University of Southern California School of Medicine, Los Angeles, CA 90033, USA; University of Iowa, 472 Psychological and Brain Sciences Building, Iowa City, IA 52242, USA; Department of Psychological Sciences, University of Missouri, , Columbia, MO 65202, USA; University of South Florida College of Nursing, Tampa, FL 33612, USA

**Keywords:** alcohol, insomnia, sleep, treatment, military

## Abstract

**Study Objectives:**

Two in five Veterans report symptoms of insomnia, with higher rates among those who drink heavily. Although Cognitive Behavioral Therapy for Insomnia (CBT-I) has demonstrated efficacy among those with Alcohol Use Disorder, abstinence is often considered a prerequisite for treatment, leaving its impact unclear among those who are actively drinking. This trial tested the efficacy of CBT-I among heavy-drinking Veterans with insomnia (#NCT03804788).

**Methods:**

Veterans from across the United States were randomly assigned to CBT-I or sleep hygiene control. Participants completed retrospective surveys and 14 sleep diaries at baseline, post-treatment, and 3-month follow-up. Primary outcomes were feasibility and insomnia severity. All other outcomes are secondary/exploratory. Intent-to-treat analyses were conducted using multilevel models.

**Results:**

Recruitment spanned June 2019 to March 2023 (*N* = 71, 80% male, *M* = 38 years). On average, we recruited 4 participants per month, with retention of 86% at post-treatment and 90% at follow-up. Of 38 CBT-I participants, 33 (87%) completed all 5 treatment sessions, and most responded to treatment (based on change in outcome scores; 22/38 at post, 27/38 at follow-up). Relative to control (*n* = 33), CBT-I participants reported large improvements in insomnia severity, both post-treatment [*d* = 1.26 (95% CI: 0.74, 1.76)] and at 3-month follow-up [*d* = 1.33 (95% CI: 0.81, 1.84)]. At follow-up, results for use of alcohol as a sleep aid [*d* = 0.66 (95% CI: 0.18, 1.14)] and sleep medication [*d* = 0.44 (95% CI: −0.03, 0.91)] also favored CBT-I.

**Conclusions:**

CBT-I is feasible among heavy-drinking Veterans and has large effects on insomnia severity. Studies testing mechanistic effects on alcohol outcomes are warranted.

**Clinical Trial Information:**

The iTAP Study for Veterans, registered on clinicaltrials.gov (#NCT03804788) on January 11, 2019: https://clinicaltrials.gov/study/NCT03804788?term=NCT03804788&rank=1

**Statement of Significance**

This study tested the feasibility and efficacy of CBT-I among heavy-drinking Veterans with insomnia to determine its potential as a mechanism of change in alcohol use outcomes. Consistent with hypotheses, CBT-I participants reported large reductions in insomnia severity that were maintained through 3-month follow-up. They also reported reductions in use of alcohol as a sleep aid, alcohol-related consequences, and use of sleep medication. CBT-I is recommended among heavy-drinking populations, regardless of abstinence.

Nearly 2 in 5 United States Veterans report symptoms of insomnia, with rates even higher among those with comorbid conditions like Alcohol Use Disorder (AUD) [1]. Rates of AUD have been increasing among Veterans in the United States [2], who demonstrate higher rates of heavy alcohol use and substance use disorders than civilians [3]. Among women Veterans between 18 and 49 years of age, the national estimated prevalence of past-month heavy drinking (4 + drinks on a single day) ranges from 27% to 43% [3]. For male Veterans between 18 and 49 years of age, the prevalence of heavy drinking (5 + drinks on a single day) is 36% to 56% [3]. Thus, a quarter to half of younger to middle-aged Veterans report heavy drinking within the past month.

Unfortunately, insomnia is especially prevalent in heavy-drinking populations, with rates between 63% and 76% among those who are actively drinking and up to 92% among those in acute alcohol withdrawal [4]. While alcohol use likely contributes to disturbed sleep [5], symptoms of insomnia may also contribute to problematic alcohol use, as 1 in 4 adults with insomnia uses alcohol specifically to help with sleep [6]. Indeed, insomnia symptoms are a consistent prospective predictor of alcohol-related problems [7–12]. Given this potentially vicious cycle between alcohol use and disturbed sleep, effective treatments for insomnia are especially needed in heavy-drinking populations, as treatment of insomnia could theoretically prevent the onset or alter the trajectory of AUD [13].

Cognitive Behavioral Therapy for Insomnia (CBT-I) is the first line of treatment for insomnia [14]. It outperforms sleep medication [15] and maintains large effects on insomnia symptoms among those with comorbid mental health conditions, including AUD [15, 16]. However, abstinence from alcohol is often considered a prerequisite for treatment [17, 18], which may be difficult for heavy-drinking adults and those with AUD. Indeed, of the 6 published trials testing the efficacy of CBT-I in heavy-drinking populations, 4 trials targeted adults in recovery from AUD (many of whom were attempting to abstain from alcohol use) [19–22] and the other 2 recruited young adults [23, 24]. While important, trials focused on those in recovery from AUD are limited because results may not generalize to those who are actively drinking. Similarly, findings for heavy-drinking young adults may not generalize to middle-aged and older adults because (a) the social contexts of drinking change from younger to older adulthood and (b) habits related to sleep and alcohol use have had more time to develop. Military service creates a particularly unique setting for sleep-related problems, as the demands of deployment, combat, and irregular sleep/wake scheduling often precipitate insomnia that persists when service members return home [25]. Perhaps for these reasons, Veterans are disproportionately affected by both insomnia and alcohol problems, particularly those who served after September 11, 2001 (post-9/11 Veterans) [1, 26, 27].

This study tested the feasibility and short-term efficacy of Cognitive Behavioral Therapy for Insomnia (CBT-I) among post-9/11 Veterans with comorbid insomnia and heavy alcohol use. A future goal of this work is to test CBT-I as a potential mechanism of change in alcohol use outcomes, in which case better understanding of the efficacy of CBT-I in reducing insomnia in this population is needed. We hypothesized that a 5-session CBT-I protocol would be feasible and acceptable among heavy-drinking Veterans with insomnia. Moreover, we hypothesized that it would outperform usual care (sleep hygiene education) in improving insomnia severity (primary outcome), both immediately post-treatment and at 3-month follow-up. All other outcomes are secondary or exploratory.

## Methods

This study was a parallel, 2-group, randomized controlled trial with a 1:1 allocation ratio. All procedures were approved by the Institutional Review Board (protocol #2014239) as well as a four-person Data Safety Monitoring Board. Reporting followed consolidated standards of reporting for randomized controlled trials (CONSORT) [28, 29].

### Participants and procedure

**Figure 1.**
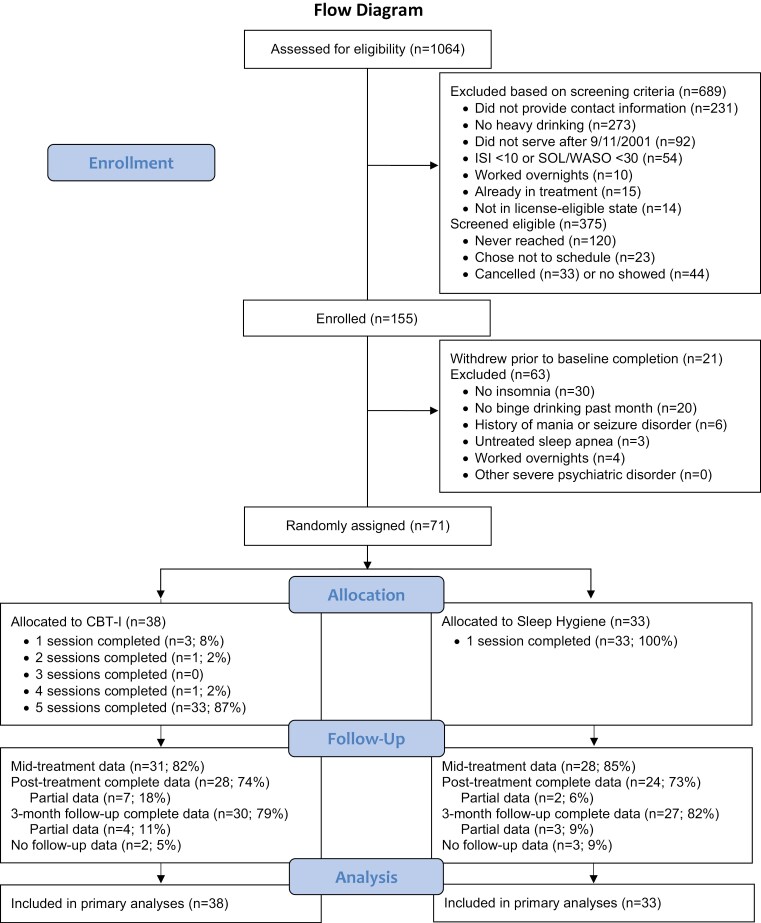
Flow diagram.

Veterans of the United States military who served after 09/11/2001 were recruited to participate in an insomnia treatment study for Veterans who drink alcohol (see Tables 1 and 2). Eligible participants (a) served after 09/11/2001, (b) reported 1 + heavy-drinking episode (≥ 4/5 drinks per occasion for women/men) or 1 + alcohol-related consequence in the past 30 days, and (c) met diagnostic criteria [30] for insomnia disorder (> 30 minutes falling asleep, staying asleep, or waking up too early on + 3 days per week for 3 + months, despite adequate opportunity and circumstances for sleep). Insomnia symptoms had to result in daytime impairment (e.g., fatigue, sleepiness, difficulty with attention or memory). Exclusion criteria included inability to provide informed consent, contraindications for sleep restriction (mania or seizures), other symptoms requiring immediate clinical attention (e.g., suicidal intent), overnight shift work, and residence in a state that does not participate in PsyPACT (see Figure 1). Although it was not listed as an exclusion criterion, no one in this trial was actively engaged in treatment for alcohol use.

**Table 1.** Participant demographics at baseline (N = 71)

	Full sample (*N* = 71)	CBT-I (*N* = 38)	Sleep hygiene (*n* = 33)	
Demographics	*n* (%) or *M* (*SD*)	*n* (%) or *M* (*SD*)	*n* (%) or *M* (*SD*)	χ^*2*^ or *t* (df)
Age	37.7 (9.3)	37.1 (7.7)	38.3 (11.1)	0.51 (56≠)
Male (vs. female) sex	57 (80%)	32 (84%)	25 (76%)	0.80 (1)
Race/ethnicity	—	—	—	—
Asian only	1 (1%)	1 (3%)	0 (0%)	1.79 (1)
American Indian only	1 (3%)	1 (3%)	0 (0%)	0.40 (1)
Black only	3 (4%)	3 (8%)	0 (0%)	0.79 (1)
Hispanic or Latinx only	3 (4%)	2 (5%)	1 (3%)	0.02 (1)
Multiracial or multiethnic	8 (10%)	2 (5%)	6 (18%)	2.95 (1)
White only	55 (78%)	29 (76%)	26 (79%)	2.44 (1)
Highest level of education	—	—	—	—
High school graduate or GED	4 (6%)	1 (3%)	3 (9%)	1.39 (1)
Some college	30 (42%)	15 (40%)	15 (46%)	0.26 (1)
College graduate	18 (25%)	14 (37%)	4 (12%)	5.70 (1)^*^
Some graduate school	9 (13%)	5 (13%)	4 (12%)	0.02 (1)
Completed graduate program	10 (14%)	3 (8%)	7 (21%)	2.69 (1)
Employment^†^	—	—	—	—
Employed	47 (66%)	24 (63%)	23 (70%)	0.34 (1)
Unemployed	15 (21%)	11 (29%)	4 (12%)	3.00 (1)
Living with disability	7 (10%)	2 (5%)	5 (15%)	1.94 (1)
Marital status	—	—	—	—
Never married	13 (18%)	9 (24%)	4 (12%)	1.58 (1)
Married or living with partner	36 (51%)	15 (39%)	21 (64%)	4.13 (1)^*^
Divorced, separated, or widowed	22 (31%)	14 (37%)	8 (24%)	1.31 (1)
Military status^‡^	—	—	—	—
Active duty	20 (28%)	9 (24%)	11 (33%)	0.81 (1)
Reserves	10 (14%)	5 (13%)	5 (15%)	0.06 (1)
Veteran	51 (72%)	27 (71%)	24 (73%)	0.02 (1)
Branch^‡^	—	—	—	—
Air Force	7 (10%)	3 (8%)	4 (12%)	0.31 (1)
Army	49 (69%)	26 (70%)	23 (70%)	.003 (1)
Marines	11 (15%)	7 (19%)	4 (12%)	1.32 (1)
Navy	4 (6%)	1 (3%)	3 (9%)	0.61 (1)
Deployed	66 (78%)	33 (87%)	22 (67%)	4.12 (1)^*^
Combat-related injury	46 (65%)	26 (68%)	20 (61%)	0.47 (1)

^*^ *p* < .05.

^†^Two participants preferred not to respond. GED = general education development test. ≠ equal variances not assumed.

^‡^Not mutually exclusive.

**Table 2.** Participant clinical characteristics at baseline (N = 71)

	Full sample (*N* = 71)	CBT-I (*n* = 38)	Sleep hygiene (*n* = 33)	
Clinical characteristic	*n* (%) or *M* (*SD*)	*n* (%) or *M* (*SD*)	*n* (%) or *M* (*SD*)	χ^*2*^ or *t* (df)
Telehealth^†^	54 (76%)	28 (74%)	26 (79%)	0.56 (1)
Alcohol Use Disorder^‡^	61 (86%)	33 (87%)	28 (85%)	0.06 (1)
Mild	23 (32%)	14 (37%)	9 (27%)	0.74 (1)
Moderate	12 (17%)	5 (13%)	7 (21%)	0.82 (1)
Severe	26 (37%)	14 (37%)	12 (36%)	.002 (1)
TLFB drinking quantity	88.1 (64.2)	94.5 (70.0)	80.9 (57.0)	0.89 (69)
TLFB heavy-drinking frequency	9.1 (8.6)	9.9 (9.1)	8.2 (8.1)	0.84 (69)
Insomnia duration (years)	12.6 (12.0)	10.6 (7.9)	14.9 (15.2)	1.53 (69)
Bedtime (diaries)^§^	11:15pm	11:10pm	11:20pm	0.82 (69)
Sleep onset latency (diaries)	44.4 (28.0)	43.7 (27.6)	45.3 (28.8)	0.24 (69)
Wake after sleep onset (diaries)	42.6 (33.9)	38.1 (34.1)	47.8 (33.5)	1.21 (69)
Waketime (diaries)^§^	6:45am	6:45am	6:50am	0.41 (69)
Total sleep time (diaries)	6.1 (1.3)	6.2 (1.2)	5.9 (1.4)	0.77 (69)
Time in bed (diaries)	8.6 (1.3)	8.6 (1.4)	8.6 (1.2)	0.02 (69)
Nightmare frequency (diaries)	2.5 (2.8)	2.8 (2.7)	2.2 (2.9)	0.86 (66)
Sleep medication (diaries)^‖^	---	---	---	---
Baseline	37 (52%)	23 (61%)	14 (42%)	2.32 (1)
Post-treatment	19 (27%)	10 (26%)	9 (27%)	0.24 (1)
Follow-up	15 (21%)	8 (21%)	7 (21%)	.001 (1)
Alcohol as a sleep aid (interview)	39 (55%)	26 (68%)	13 (39%)	3.74 (1)
Alcohol as a sleep aid (diaries)	---	---	---	---
Baseline	25 (35%)	16 (42%)	9 (27%)	1.70 (1)
Post-treatment	8 (11%)	5 (13%)	3 (9%)	0.11 (1)
Follow-up	9 (13%)	5 (13%)	4 (12%)	0.03 (1)
Substance use, past month	---	---	---	---
Cigarettes	14 (20%)	11 (29%)	3 (9%)	4.40 (1)*
Smokeless tobacco	13 (18%)	7 (18%)	6 (18%)	.001 (1)
Cannabis	25 (35%)	15 (39%)	10 (30%)	0.65 (1)
Symptoms of depression (PHQ)	10.1 (4.7)	10.3 (5.0)	9.8 (4.5)	0.42 (67)
Trauma exposure (interview)	60 (85%)	34 (90%)	26 (79%)	1.54 (1)
Symptoms of PTSD (PCL-5)	25.2 (18.3)	26.8 (18.9)	23.3 (17.7)	0.83 (67)
Symptoms of anxiety (GAD-7)	8.3 (5.1)	7.9 (5.0)	8.8 (5.1)	0.78 (68)
Sleep apnea, high risk (STOP)	17 (24%)	10 (26%)	7 (21%)	0.98 (1)

^*^ *p* < .05.

^†^Coded “yes” if any sessions were completed remotely.

^‡^Diagnosed via the MINI-7.0.2.

^§^Participants reported in minutes in military time; translated to AM/PM and 5-minute intervals for ease of interpretation.

^‖^As reported on the clinical interview or sleep diaries.

GAD-7 = Generalized Anxiety Disorder scale [63]. PCL-5 = PTSD Checklist for DSM-5 [64]. PHQ-9 = Patient Health Questionnaire [65]. Rx = prescription. STOP = sleep apnea STOP-BANG screening instrument [66]. TLFB = Timeline Followback interview, drinking past 30 days [42].

From June 2019 through February 2020, participants were recruited via local community advertisement, and all procedures were conducted in person. From March 2020 through January 2021, recruitment was paused due to COVID-19. In February 2021, we re-initiated recruitment with remote protocols, allowing for broader recruitment of Veterans across the United States. In April 2022, we contracted with the digital marketing company BuildClinical to assist with recruitment. In all cases, interested individuals completed an online screening form and provided written informed consent before completing a retrospective online survey. They then completed a semi-structured clinical interview with a trained research assistant. The semi-structured interview assessed medical and treatment history; current sleep pattern; symptoms of insomnia, circadian rhythm disorders, sleep apnea, restless leg syndrome, non-REM sleep arousals, nightmare disorder, non-REM sleep arousal disorders, REM sleep behavior disorder, and narcolepsy; as well as the depression, mania, post-traumatic stress disorder, AUD, and psychosis sections of the MINI International Neuropsychiatric Interview for DSM-5 [31] and the Columbia Suicide Severity Rating Scale [32]. Those who were eligible based on the clinical interview were scheduled to complete 14 consecutive days of morning sleep diaries, which were used to confirm diagnosis of insomnia.

Participants completed follow-up assessments at the end of treatment (“post-treatment”) and 3 months after the end of treatment. Each follow-up included a retrospective survey and 2 weeks of morning sleep diaries. They received up to $300 for completing all aspects of the study.

### Interventions

Both interventions were delivered individually in 30–60 minute sessions by trained graduate students, either in-person or via telehealth (secure Zoom video; see Table 2). In-person encounters occurred in private rooms in the principal investigator’s clinical research laboratory. All clinical encounters were audiotaped and reviewed by the supervising licensed clinical psychologist (MBM) for ongoing supervision and training; and the supervising psychologist, in turn, was supervised by a Diplomate of Behavioral Sleep Medicine (CSM). The therapy manual and handouts are available from the first author upon request.

#### Cognitive behavioral therapy for insomnia (CBT-I).

CBT-I is a multidimensional treatment that targets the thoughts and behaviors that perpetuate insomnia symptoms over time [33]. The cognitive components of therapy empower patients to challenge their automatic thoughts and reactions to poor sleep [34], and the behavioral components focus on matching time in bed to sleep ability [18]. Therapists followed a 5-session treatment protocol. All sessions began with a review of sleep diary data. After diary review, Session 1 transitioned to education on the development of insomnia and treatment rationale. When discussing perpetuating factors for insomnia, therapists spent ~5 minutes reviewing alcohol’s acute effects on sleep; specifically, heavy drinking before bedtime may reduce sleep onset latency (SOL) in the short-term, but (a) most people develop a tolerance to the sleep-promoting effects of alcohol within a couple nights [35] and (b) it tends to disrupt sleep in the second half of the night [36]. They then reviewed a sleep hygiene handout identical to the handout provided to the sleep hygiene control group. Sessions 2 through 5 began with review of diaries and treatment adherence. Specific content included stimulus control and sleep restriction (Session 2), relaxation techniques (Session 3), cognitive strategies (Session 4), and insomnia relapse prevention (Session 5). Therapy guidelines were consistent with the VA’s CBT-I therapy manual; specifically, minimum of 5 hours in bed, restrict to average total sleep time, and increase time in bed when sleep efficiency ≥ 85% and sleep need is high [37].

#### Sleep hygiene.

 To model “usual care,” sleep hygiene participants met with therapists in a single session to discuss the sleep hygiene recommendations on the National Sleep Foundation’s website in 2018. Specific recommendations included limiting daytime naps to 30 minutes; avoiding caffeine, nicotine, alcohol use, strenuous workouts, and rich or fatty foods before bedtime; getting sunlight during the day; establishing a bedtime routine; and creating a relaxing bedroom environment. Participants chose 1 or 2 recommendations to prioritize independently over the next 5 weeks.

#### Brief alcohol intervention.

Given the heavy-drinking nature of the sample, a brief evidence-based [38] alcohol intervention was delivered to participants in both conditions, alongside the sleep hygiene recommendation to avoid alcohol before bedtime. Specifically, therapists presented personalized normative feedback comparing the number of drinks participants consumed each week to (a) the number of standard drinks that same-sex Veterans consume and (b) the number of drinks they reported believing same-sex Veterans consume [39]. Therapists were trained to present the information in a non-judgmental style, to elicit participants’ reactions to the content, and to help them brainstorm why most people overestimate the norm [40]. They were also instructed to spend no more time on this content than the participant wanted to spend. Discussions of the feedback averaged 3–4 minutes.

#### Treatment integrity.

Consistent with recommendations [41], interventionists received initial training via mock sessions and audiotaped all treatment sessions for ongoing supervision and feedback. The principal investigator reviewed all tapes for therapists’ first participant and tapered down to ~10% of remaining tapes to ensure treatment integrity. Participants received a workbook of treatment materials to facilitate comprehension, and therapists reviewed and addressed non-adherence each week to encourage enactment. Fifty audiotapes were randomly selected for treatment integrity coding, which was completed using a checklist of treatment elements (0%–100% integrity rating).

#### Protocol modifications.

COVID-19 resulted in the following protocol modifications after trial commencement: (1) broadened inclusion criteria, such that participants could report either heavy drinking or alcohol-related consequences in the past month and had to serve but not necessarily deploy after 9/11/2001; (2) administration of assessments and treatment via telehealth; (3) inclusion of Veterans across all United States; (4) inconsistent delivery of Phillips Respironics Spectrum Plus actiwatch devices; and (5) discontinued collection of Timeline Followback [42] data at follow-up. Actigraphy data were not analyzed due to insufficient data (baseline *n* = 28; follow-up *n* = 11). In addition, because our pre-registration specified more primary outcomes than recommended [29], we modified analyses to focus on insomnia severity as the primary outcome, as this is the primary proposed mechanism of CBT-I effects on alcohol outcomes (as noted in our previously-published conceptual model [43]) and was the outcome used to determine sample size.

### Measures

#### Treatment satisfaction and credibility.

At the end of treatment, participants completed the 8-item Client Satisfaction Questionnaire (e.g., *How would you rate the quality of insomnia treatment you received?*) [44]. Responses ranged from 1 (*poor*) to 4 (*excellent*). This measure has been validated among individuals with substance use disorders [45] and demonstrated strong internal consistency in this sample (α=.94).

#### Insomnia severity.

Insomnia was diagnosed via clinical interview, retrospective self-report, and prospective sleep diaries. At each assessment (baseline, post-treatment, 3 months), symptoms of insomnia were assessed retrospectively using the Insomnia Severity Index (ISI) [46]. The ISI is a 7-item measure of sleep problems in the past 2 weeks. Responses range from 0 to 4, with higher scores indicating more severe insomnia. Scores ≥ 10 indicate clinically significant insomnia in community samples [46]. For descriptive purposes, participants who reported ≥ 8-point decreases on the ISI were categorized as treatment responders, and those with scores < 8 were categorized as remitted [46]. Internal consistency of the ISI in this sample was good (α=.87). The pre-specified criterion for continuing with a larger mechanistic trial was evidence that CBT-I significantly reduces insomnia severity in this population.

Sleep diaries were completed in 14-day bursts at baseline, post, and 3-month follow-up. Following consensus recommendations [47], participants reported the time they got into bed, how long it took to fall asleep (SOL), minutes awake in the middle of the night (WASO), time of their final awakening, time they got out of bed, and sleep quality (0 *very poor* to 4 *very good*). Total sleep time was calculated by subtracting SOL + WASO from the time elapsed between time asleep and time of final awakening. Sleep efficiency was calculated by dividing total sleep time by time in bed (0%–100%). Participants also indicated (0 = no, 1 = yes) if they had used a sleep medication, the number of standard drinks they consumed, and if they had used alcohol specifically to help with sleep.

#### Alcohol use.

AUD was diagnosed via clinical interview (Mini International Neuropsychiatric Interview [31], Version 7.0.2). Drinking quantity and alcohol-related consequences were assessed at baseline, post-treatment, and 3-month follow-up. The Daily Drinking Questionnaire (DDQ) [48] assessed the typical number of standard drinks consumed on each day of a typical week (Sunday through Saturday) in the past month. The outcome of interest was the total number of drinks consumed in a typical week. This variable was strongly correlated with total drinks reported on the Timeline Followback [42] [*r*(71) = 0.79, *p* < .001].

The Brief Young Adult Alcohol Consequences Questionnaire [49] was used to assess alcohol-related consequences in the past month. Participants indicate (0 = no, 1 = yes) if they had experienced 24 consequences (e.g., hangover, embarrassment, interpersonal conflict) as a result of drinking in the past month. Responses were summed to create a total score.

### Sample size determination

A-priori power analyses used G-Power to estimate the necessary sample size for a repeated measures analysis of variance (ANOVA) because this would overestimate the sample size needed for the proposed multilevel models. Based on previous trials, expected effect sizes for insomnia severity were large [50, 51] and for alcohol outcomes were small to medium [52]. Using repeated measures ANOVA, within-between interaction (α=.05, power = .95, groups = 2, repetitions = 4, correlation = .40), the sample size needed to detect a small effect (*f* = .10) was 260; a medium effect (*f* = .25) was 44; and a large effect (*f* = .40) was 18. Accounting for attrition, we aimed to recruit 68 participants to obtain a final sample of 44 participants.

The principal investigator monitored adverse events and discussed issues with the full research team as they arose. Early stopping rules included plans to discontinue the trial if evidence emerged that CBT-I had either (a) serious adverse effects or (b) such beneficial effects that denial to the control group would be unethical. We also planned to discontinue if it seemed unlikely that study aims would be achieved.

### Randomization

Participants were randomly assigned to CBT-I or sleep hygiene control following simple randomization procedures. The principal investigator (MBM) generated the random allocation sequence (1:1 ratio) using a computerized random number generator and then gave it to the project coordinator (who was not involved in clinical or enrollment decisions) to conceal. The principal investigator did not retain a copy of the allocation sequence or access it during recruitment. Graduate student assessors determined eligibility, in consultation with supervising psychologists MBM and CSM as needed (e.g., if diagnosis of insomnia was unclear). At the first “sleep coach” meeting, the graduate student therapist informed participants of their eligibility and confirmed their continued interest in the study. If participants confirmed interest, the therapist would step out of the room and ask the project coordinator for the participant’s random assignment. The project coordinator, who stored the allocation on their personal secure network server, would check the allocation sequence and record group assignment. Therapists then enrolled participants in the trial.

### Masking

Participants were told that they would be randomly assigned to 1 of 2 “treatment” groups: 1 that occurred in 1 session or 1 that occurred in 5 sessions. Due to the nature of the treatment and design, participants were aware of their group assignment, as were study therapists and the statistical analyst.

### Data screening and analysis

Descriptive statistics and chi-square analyses were used to contextualize feasibility, acceptability, response to treatment (ISI change-scores ≥ 8), and remission from insomnia (ISI scores < 8) [46]. Multilevel models were used to account for nesting of assessments within persons. Analyses were intent-to-treat, meaning participants in treatment and control groups were categorized and included in analyses according to random assignment, regardless of treatment completion. Models were conducted in SAS 9.4 (SAS Institute, 2014) using PROC MIXED or PROC GLIMMIX with restricted maximum likelihood estimation, an unstructured covariance structure, and a person-level random intercept. For count outcomes (e.g., alcohol-related consequences), generalized linear models were conducted using a negative binomial distribution. For outcomes involving the number of days an event occurred (i.e., days with use of alcohol to help with sleep, days with use of sleep medication), logistic models with a binomial distribution were conducted with the outcome in event/trial format (i.e., number of events/total number of diary days). Separate models were conducted for each outcome (see Table 5). Predictors included time (treated as a single categorical predictor, with baseline as the reference time point), treatment group (0 = sleep hygiene, 1 = CBT-I), and group-by-time interactions. An alpha of .05 was used for all pre-registered outcomes, which focused on planned comparisons from baseline to post and follow-up [53]. Effect sizes were calculated as the observed mean difference between groups, divided by the pooled baseline standard deviation [Cohen’s *d* = 0.20 small, 0.50 medium, 0.80 large; see Tables 3 and 4]. For count outcomes (alcohol-related consequences, drinks per week) robust Cohen’s *d*s were calculated [54]. No ancillary analyses were conducted.

**Table 3.** Means and effect sizes for sleep outcomes (N = 71)

	CBT for insomnia			Sleep hygiene			Effect size
	*n*	*M*	*SD*	*n*	*M*	*SD*	Cohen’s *d* [95% CI]
*Primary*							
Insomnia severity							
Baseline	38	17.29	4.39	33	16.88	4.15	
Mid-treatment	31	10.71^W^	5.26	28	13.07^W^	5.28	
Post-treatment	34	7.24^WB*^	4.83	26	12.27^WB*^	4.57	1.26 [0.74, 1.76]
3mo follow-up	33	6.97^WB*^	5.84	30	12.30^WB*^	5.14	1.33 [0.81, 1.84]
*Secondary*							
Diary SQ							
Baseline	38	1.92	0.60	33	1.73	0.65	
Post-treatment	32	2.49^WB^	0.52	24	2.13^WB^	0.87	0.29 [−0.18, 0.76]
3 mo follow-up	26	2.53^WB^	0.63	23	2.14^WB^	0.93	0.32 [−0.15, 0.79]
Diary SE							
Baseline	38	71.94	11.84	33	68.82	12.07	
Post-treatment	32	87.89^WB*^	6.39	24	77.89^WB*^	8.84	0.57 [0.09, 1.04]
3 mo follow-up	26	87.10^WB*^	9.57	23	79.90^WB*^	10.57	0.34 [−0.13, 0.81]
Sleep med days (%)							
Baseline	38	30.92	36.23	33	23.15	35.49	
Post-treatment	32	14.04^WB*^	30.71	24	28.44^B*^	41.56	0.49 [0.02, 0.96]
3 mo follow-up	26	11.55^WB*^	28.18	23	20.36^B*^	38.35	0.44 [−0.03, 0.91]
*Exploratory*							
Diary SOL							
Baseline	38	43.69	27.64	33	45.31	28.78	
Post-treatment	32	16.43^WB*^	10.12	24	30.07^WB*^	24.37	0.42 [−0.05, 0.89]
3 mo follow-up	27	19.01^W^	21.07	23	26.77^W^	18.35	0.22 [−0.25, 0.68]
Diary WASO							
Baseline	38	38.10	34.13	33	47.80	33.47	
Post-treatment	32	15.08^WB*^	14.43	24	38.07^WB*^	29.90	0.39 [−0.08, 0.86]
3 mo follow-up	26	18.69^W^	19.78	23	26.15^W^	22.26	−0.07 [−0.53, 0.40]
Diary TST							
Baseline	38	6.17	1.23	33	5.93	1.35	
Post-treatment	32	6.60	1.27	24	6.69^W^	1.17	0.25 [−0.22, 0.72]
3 mo follow-up	26	6.86^W^	1.27	23	6.52^W^	1.29	−0.08 [−0.55, 0.39]

Reports based on all available data; nothing imputed or listwise deleted. Cohen’s *d* calculated from observed means and SDs as the mean difference between groups, divided by the pooled baseline standard deviation; interpreted as 0.20 small, 0.50 medium, and 0.80 large. *group-by-time interaction was significant. ^B^pairwise comparison indicated significant (*p* < .05) between-group difference. ^W^pairwise comparison indicated significant (*p* < .05) within-group change from baseline. Mo = month. SE = sleep efficiency. SOL = sleep onset latency. SQ = sleep quality. TST = total sleep time. WASO = wake after sleep onset.

**Table 4.** Means and effect sizes for alcohol outcomes (N = 71)

	CBT for Insomnia			Sleep Hygiene			Effect Size
	*n*	*M*	*SD*	*n*	*M*	*SD*	Cohen’s *d* [95% CI]
Drinks per week^a^							
Baseline	38	18.63	15.76	33	17.94	15.26	
Mid-treatment	30	17.23	15.63	27	18.70	14.27	
Post-treatment	31	15.29^W^	14.73	26	12.00^M^	9.84	−0.45 [−0.92, 0.02]
3mo follow-up	33	15.12^W^	21.96	30	15.73	15.89	0.09 [−0.37, 0.56]
Consequences^a^							
Baseline	38	7.92	5.34	33	8.45	6.49	
Post-treatment	31	2.45^WB^^*^	3.25	24	4.46^WB^^*^	4.76	0.45 [−0.02, 0.92]
3mo follow-up	32	3.31^WB^	4.20	29	5.28^WB^	4.35	0.55 [0.08, 1.03]
Alcohol craving							
Baseline	38	9.29	5.63	33	9.70	6.82	
Post-treatment	28	6.46^W^	5.91	24	7.62	6.74	0.12 [−0.35, 0.59]
3mo follow-up	30	7.30^W^	7.18	27	7.04^W^	5.96	−0.11 [−0.57, 0.36]
Alcohol sleep days (%)							
Baseline	38	16.71^B^	27.86	33	6.73^B^	16.56	
Post-treatment	32	3.22^W^^*^	8.60	24	6.89	22.94	0.56 [0.08, 1.03]
3 mo follow-up	26	2.24^WB^^*^	4.88	23	9.12^B^^*^	26.17	0.66 [0.18, 1.14]

*Note.* Reports based on all available data; nothing imputed or listwise deleted.

Cohen’s *d* calculated from observed means and SDs as the mean difference between groups, divided by the pooled baseline standard deviation; interpreted as 0.20 small, 0.50 medium, and 0.80 large.

^*^group-by-time interaction was significant.

^a^Robust Cohen’s *d* reported for effect size (Algina et al, 2005).

^W^pairwise comparison indicated significant (*p* < .05) within-group change from baseline.

^M^within-group least square means = −0.32 (CI: −0.66, 0.02), *p* = .07.

^B^pairwise comparison indicated significant (*p* < .05) between-group difference.

CI = confidence interval. Mo = month.

**Table 5.** Inferential statistics (n = 38 CBT-I and n = 33 control).

	Group		Group × post		Group × follow-up	
	Est [95% CI]	*P*	Est [95% CI]	*P*	Est [95% CI]	*P*
*Sleep*						
Insomnia severity§	0.41 [−1.91, 2.73]	.727	−5.33 [−7.62, −3.03]	<.001	−5.31 [−7.55, −3.06]	<.001
Diary sleep quality	0.18 [−0.14, 0.51]	.265	0.25 [−0.09, 0.58]	.149	0.20 [−0.15, 0.55]	.268
Diary sleep efficiency	3.12 [−1.68, 7.92]	.199	7.18 [2.05, 12.32]	.007	6.19 [0.83, 11.54]	.024
Diary SOL	−1.62 [−12.56, 9.31]	.769	−14.88 [−27.98, −1.78]	.026	−8.86 [−22.42, 4.71]	.198
Diary WASO	−9.70 [−22.74, 3.34]	.143	−12.02 [−26.23, 2.19]	.096	−3.56 [−18.38, 11.26]	.635
Diary TST	0.24 [−0.36, 0.83]	.436	−0.33 [−0.93, 0.27]	.275	0.26 [−0.37, 0.88]	.416
Sleep med days^b^	1.20 [−0.44, 2.84]	.149	−3.06 [−3.98, −2.15]	<.001	−3.15 [−4.25, −2.05]	<.001
*Alcohol*						
Drinks per week^a^	0.01 [−0.45, 0.47]	.969	−0.03 [−0.50, 0.44]	.914	−0.27 [−0.73, 0.19]	.236
Alcohol consequences^a^	−0.06 [−0.52, 0.41]	.812	−0.66 [−1.23, −0.10]	.022	−0.51 [−1.03, 0.01]	.056
Alcohol craving	−0.41 [−4.78, −0.93]	.789	−1.15 [−3.89, 1.60]	.410	0.64 [−2.01, 3.29]	.633
Alcohol sleep aid^b^	1.53 [0.21, 2.84]	.023	−2.51 [−3.68, −1.35]	<.001	−3.29 [−4.49, −2.09]	<.001

*Note.* §primary outcome.

^a^Generalized linear model conducted using a negative binomial distribution.

^b^Generalized logistic model using a binomial distribution with the outcome in event/trial format.

CI = confidence interval; Med = medication; SOL = sleep onset latency; WASO = wake after sleep onset; TST = total sleep time.

*F*-values [*p*] for the interaction of group by (linear) time: insomnia severity = 10.12 [< .001], sleep quality = 1.20 [.31], sleep efficiency = 4.57 [.01], days using sleep medication = 27.63 [< .001], drinks per week = 0.78 [.50], alcohol consequences = 3.40 [.04], alcohol craving = 0.80 [.45], days using alcohol as a sleep aid = 18.11 [< .001].

## Results

Primary outcomes were feasibility (characterized using recruitment and retention rates), acceptability (treatment satisfaction), and insomnia severity. All other outcomes were secondary or exploratory (see Table 3). Means, standard deviations, and effect sizes for between-group planned comparisons from baseline to post and follow-up are presented in Tables 3 (sleep outcomes) and 4 (alcohol outcomes), and inferential statistics are presented in Table 5. For outcomes with significant group-by-time interactions, least square means (LSM) estimates representing the between-group effects at each time point are reported in text.

### Primary outcomes

Recruitment and retention are depicted in Figure 1. Recruitment, which spanned June 2019 to March 2023, was interrupted by the COVID-19 pandemic. However, in the final year of recruitment, 87 participants completed baseline procedures (7–8/month) and 48 were randomized (4/month). Recruitment ended when the target sample size had been enrolled. Of the 155 participants who completed baseline, 71 met all eligibility criteria (see Tables 1 and 2). Recruitment was successful in reaching heavy-drinking Veterans: all participants reported heavy episodic drinking (≥4/5 drinks for women/men) in the past month at baseline, 51/71 (72%) reported heavy episodic drinking every month for the past year, and 48/71 (68%) reported heavy episodic drinking on a weekly basis.

Participants were randomized to single-session sleep hygiene (*n* = 33) or 5-session Cognitive Behavioral Therapy for Insomnia (CBT-I; *n* = 38). Of 38 CBT-I participants, 33 (87%) completed all 5 treatment sessions (see Figure 1), indicating good treatment retention. The average number of weeks for treatment completion was 5.54 (*SD* = 1.77), and the average integrity rating for therapist adherence to the protocol was 97.50% (*SD* = 6.00; range 70%–100%). CBT-I participants reported average satisfaction ratings from 2–4 (1–4 scale), with an average of 3.59 (*SD* = 0.66). Overall, 97% of participants rated CBT-I as “good” or “excellent,” and all agreed they would recommend CBT-I to a friend in need of similar help.

Relative to sleep hygiene, CBT-I participants reported large reductions in insomnia severity from baseline to post [LSM = −4.92 (CI: −7.37, −2.46), *p* < .001] that were maintained at 3-month follow-up [LSM = −4.90 (CI: −7.31, −2.49), *p* < .001].

### Secondary and exploratory outcomes

Changes in sleep quality did not differ significantly between groups (see Tables 3 and 5). However, CBT-I participants reported greater improvements in sleep efficiency than control at post [LSM = 10.30 (CI: 5.05, 15.56), *p* < .001] and follow-up [LSM = 9.31 (CI: 3.83, 14.78), *p* = .001]. Given this significant finding for sleep efficiency, we explored CBT-I effects on SOL, wake after sleep onset (WASO), and total sleep time. Immediately after treatment, CBT-I participants reported greater reductions in both SOL [LSM = −16.50 (CI: −28.66, −4.34), *p* = .008] and WASO [LSM = −21.72 (CI: −36.04, −7.40), *p* = .003] than sleep hygiene participants (although the overall group-by-time interaction was not significant for WASO at post; see Table 5). At 3-month follow-up, CBT-I values were still improved from baseline but no longer significantly different from sleep hygiene for either SOL [LSM = −10.48 (CI: −23.14, 2.18), *p* = .104] or WASO [LSM = −13.26 (CI: −28.19, 1.67), *p* = .081]. Change in total sleep time did not differ between groups at post-treatment [LSM = −0.09 (CI: −0.74, 0.55), *p* = .771] or follow-up [LSM = 0.49 (CI: −0.18, 1.16), *p* = .150].

The percentage of participants reporting use of sleep medication in each group at each time point is depicted in Table 2, and the percentage of days they reported using sleep medication is depicted in Table 3. Although the percentage of CBT-I and control participants who reported using sleep medication was similar at post and follow-up (see Table 2), CBT-I participants reported greater decreases in frequency of sleep medication use than control participants at both post [LSM = −1.86 (CI: −3.60, −0.12), *p* = .03] and follow-up [LSM = −1.95 (CI: −3.79, −0.11), *p* = .04].

There were no significant between-group differences in change in drinking quantity over time; however, results for alcohol-related consequences favored CBT-I (see Tables 4 and 5). Relative to control, CBT-I participants reported greater decreases in alcohol-related consequences at post [LSM = −0.72 (CI: −1.29, −0.15), *p* = .01] that were arguably maintained at follow-up [LSM = −0.56 (CI: −1.09, −0.03), *p* = .04 but group-by-time interaction *p* = .056; see Table 5]. Both groups reported reductions in alcohol craving over time, with no significant group differences at post or follow-up (see Tables 4 and 5). The percentage of participants reporting use of alcohol as a sleep aid is presented in Table 2, and the percentage of days they reported using alcohol as a sleep aid is in Table 4. There were group differences in the percentage of days using alcohol as a sleep aid (see Table 5): CBT-I participants reported more frequent use of alcohol for sleep at baseline [LSM = 1.53 (CI: 0.21, 2.84), *p* = .02], did not differ from sleep hygiene at post [LSM = −0.99 (CI: −2.55, 0.58), *p* = .21], and reported less frequent use of alcohol for sleep at follow-up [LSM = −1.76 (CI: −3.36, −0.17), *p* = .03].

### Clinical significance

Clinical significance was tested assuming those lost to follow-up did not respond/remit. At post-treatment, a significantly greater proportion of participants in the CBT-I than sleep hygiene group responded to treatment [22/38 (58%) CBT-I vs 7/33 (21%) control; χ^2^(1) = 9.84, *p* = .002] and were in remission based on insomnia severity scores [19/38 (50%) CBT-I vs 3/33 (9%) control; χ^2^(1) = 13.82, *p* < .001]. This pattern was maintained at 3-month follow-up: a greater proportion of CBT-I participants responded to treatment [27/38 (71%) vs 11/33 (33%); χ^2^(1) = 10.10, *p* = .001] and were categorized as in remission [23/38 (61%) vs 3/33 (9%); χ^2^(1) = 20.13, *p* < .001].

### Adverse events

One adverse event occurred: 1 participant reported unexpected distress in response to the PTSD and moral injury assessment items, which were part of the larger assessment battery. No other serious or unexpected adverse events were identified.

## Discussion

Consistent with hypotheses, Cognitive Behavioral Therapy for Insomnia (CBT-I) was feasible and acceptable among heavy-drinking Veterans with insomnia. Recruitment and retention rates indicate clear feasibility not only of recruiting Veterans at high risk for alcohol-related problems (86% of whom met criteria for AUD) in insomnia treatment trials, but also retaining them in research for at least 3 months. Treatment was also well-received, with 4 out of 5 participants completing all 5 treatment sessions, nearly all rating it as “good” or “excellent,” and all recommending it to others.

CBT-I was also associated with large, sustained improvements in insomnia symptoms, with 71% of participants experiencing at least minimally important differences in insomnia and 61% in remission from insomnia (based on ISI < 8) at 3-month follow-up. These are clinically meaningful improvements in insomnia that match or exceed those found in CBT-I trials that exclude for active substance use [55]. Indeed, improvements were meaningful enough that they were accompanied by significant reductions in use of sleep medication. These data, in combination with 2 previous trials documenting improvements in insomnia among heavy-drinking young adults [23, 24], challenge the idea that individuals must abstain from alcohol to benefit from CBT-I [17]. Not only is CBT-I highly needed among those who drink heavily [4], it retains large effects on insomnia in this population [15, 23, 24].

Results of this trial support future research testing CBT-I (and associated improvements in insomnia) as a mechanism of change in alcohol use outcomes. Although CBT-I did not reduce drinking quantity relative to single-session sleep hygiene control, it did reduce the number of alcohol-related consequences that Veterans experienced at the end of treatment, and the size (but perhaps not statistical significance) of this effect was maintained at 3-month follow-up. This is now the third trial to include alcohol-related consequences as an outcome, and it is the third to find that CBT-I is linked (directly or indirectly) to reductions in alcohol-related harm [22, 23]. We are aware of only one study demonstrating CBT-I effects on drinking: Verlinden and colleagues (2023) [24] found that, relative to education control, digital CBT-I reduced both quantity and frequency of alcohol use among young adults reporting heavy drinking on a weekly basis. To increase generalizability, our study included Veterans who reported heavy drinking 1 + time in the past month (as opposed to heavy drinking every week). Based on these findings, future studies may need to recruit participants who drink heavily on a more regular basis to document treatment effects on drinking patterns.

Consistent with the idea that CBT-I may have promise as a mechanism of change in alcohol outcomes, findings for use of alcohol as a sleep aid suggest that CBT-I may also remove one motive for alcohol use (i.e., to help with sleep). However, effects are difficult to interpret because CBT-I participants were more likely than sleep hygiene participants to report using alcohol as a sleep aid at baseline. Given this baseline group difference, it is unclear if the CBT-I reduction in number of alcohol-to-sleep days represents true change or regression to the mean. However, in the CBT-I group, 16 participants used alcohol to help with sleep on ~40% of days at baseline, and only 5 of those participants used alcohol to help with sleep on ~20% and then ~12% of days at post and follow-up, respectively. This study was underpowered to test CBT-I effects on this group specifically. Studies testing the efficacy of CBT-I in reducing alcohol use among those who use alcohol for sleep are encouraged.

Consistent with studies indicating that CBT-I improves diary-assessed sleep parameters in heavy-drinking samples [19–21], we found a significant effect on diary-assessed sleep efficiency that was sustained at 3-month follow-up. Exploratory analyses indicate that this may be due to CBT-I effects on both SOL and WASO (at least at post-treatment). As is typical in studies involving sleep restriction, total sleep time did not improve significantly in the CBT-I group at post, but did improve (relative to baseline) at follow-up. Given these findings and the magnitude of treatment effects on insomnia symptoms, it seems counterintuitive that we did not observe a treatment effect on diary-assessed sleep quality. At least one previous study found significant reductions in diary-assessed sleep quality as a result of CBT-I [23], but others have not found effects [20] or have not included it as an outcome [21, 22, 24]. Another study found CBT-I effects on diary sleep quality using a 0–10 (rather than 0–4) scale [19]. We speculate that the restricted range of the single sleep quality item (albeit a consensus recommendation [47]) may have limited the variability needed to find effects on this outcome.

### Clinical implications

While fully-powered randomized controlled trials are needed to validate and test the generalizability of CBT-I effects on alcohol use outcomes, evidence to date are clear that CBT-I retains efficacy in reducing insomnia among those actively engaged in heavy drinking [23, 24].

CBT-I is also more effective than sleep medication among those with AUD [15] and reduces insomnia when delivered as an adjunct to alcohol treatment [22]. Regardless of CBT-I effects (or non-effects) on substance use, the ubiquity of insomnia among those with AUD (70%–90%) [4] and efficacy of CBT-I in treating insomnia within this population supports wide dissemination of CBT-I in alcohol prevention and treatment settings. Importantly, this study and others have demonstrated efficacy of CBT-I when delivered via telehealth and fully online web-based programming [22, 24, 55]. Given the prevalence and impact of insomnia among Veterans and the rurality of this population, continued research on novel ways to disseminate CBT-I is needed.

### Limitations and future directions

This trial tested the feasibility and efficacy of CBT-I in a high-need population that tends to be underrepresented in clinical trials (military/Veterans who are actively engaged in heavy alcohol use). However, findings should be interpreted in the context of limitations. First, data collection spanned the COVID-19 pandemic, which changed the way people drink [56] and required modifications to the protocol. For example, most treatment sessions were conducted via telehealth. We were also unable to successfully implement remote delivery and collection of actigraphy data. As a result, all data were collected via self-report. Self-report measures of drinking are strongly correlated with alcohol biomarkers [57], self-report is the recommended method of assessment for insomnia [58], and subjective sleep measures are more strongly associated with future alcohol use than objective measures [59]. However, it would be useful in future trials to test the extent to which CBT-I changes objective measures of sleep and sleep architecture.

The trial was also limited by suboptimal allocation concealment and lack of true masking (“open label” design). Specifically, the investigator who generated the random allocation sequence also supervised the graduate students who enrolled participants in the trial. Although the investigator did not retain a copy of the allocation sequence or access it throughout the trial, it is best practice for the person who generates the allocation sequence to be uninvolved in participant enrollment, as knowledge of treatment assignment can result in selection bias [29]. Similarly, participants were aware of their group assignment (to 1 or 5 sessions of “treatment”). This design was chosen to model usual care (i.e., primary care providers might offer sleep hygiene recommendations to patients with sleep complaints) but may result in reporting bias.

Two aspects of the control group are also worth noting. First, the control condition was not matched with CBT-I for time and content. We chose this design intentionally because the aim of this line of research is mechanistic. Specifically, while traditional treatment studies aim to determine if one intervention is better than another (in which case a time-matched control is important), the aim of this work is to determine if change in insomnia leads to improvements in alcohol outcomes. The success of such an aim depends on production of the largest possible between-group difference in outcome [60, 61], in which case we chose the least stringent control group deemed ethical to use (single-session sleep hygiene education). While this design cannot determine if improvements in insomnia are due to CBT-I or non-specific therapy effects, previous trials have demonstrated superiority of CBT-I over sleep-related control groups that are matched for time [20, 62]. At the same time, because the sleep hygiene group was designed to model “usual care,” sleep hygiene participants received some sleep recommendations that overlap with CBT-I. For example, they were encouraged to limit daytime naps, get sunlight during the day, and avoid bright light at night. Theoretically, consistent adherence to these recommendations could lead to improvements in sleep, which may have limited between-group differences.

Finally, data were collected from a relatively small, homogeneous sample of heavy-drinking Veterans. Although CBT-I has demonstrated efficacy across a range of samples [16], findings from this study may not generalize to all populations. Inclusion of those with a wide range of comorbidities, while important for external validity, also may have obscured some outcomes. We encourage future studies testing efficacy across diverse racial/ethnic groups, sex/genders, and those with other substance use disorders.

## Conclusion

Cognitive Behavioral Therapy for Insomnia (CBT-I) is feasible and highly effective in reducing insomnia among heavy-drinking Veterans. Rather than asking participants to abstain from drinking, we strongly encourage providers to use CBT-I as an opportunity to challenge participants’ beliefs about the way that alcohol impacts their sleep. Findings support a future, fully-powered mechanistic trial testing change in insomnia as a mechanism of change in alcohol use outcomes, perhaps focused on those who report weekly heavy drinking or use of alcohol specifically to help with sleep.

## Data sharing

The first author will make de-identified participant data available to other qualified investigators who submit a signed data access agreement. The request will be evaluated by the PI to ensure that it meets reasonable demands of scientific integrity.
